# Direct cost analysis of the second year post-allogeneic hematopoietic stem cell transplantation in the Bone Marrow Transplant Centre of Tunisia

**DOI:** 10.1080/20016689.2017.1335161

**Published:** 2017-06-15

**Authors:** Myriam Razgallah Khrouf, Leila Achour, Asma Thabti, Mohamed Ali Soussi, Nour Abdejelil, Olfa Lazreg, Chema Drira, Aida Zahaf, Saloua Ladab, Tarek Ben Othman

**Affiliations:** ^a^ Service Pharmacie, Centre National de Greffe de Moelle Osseuse, Tunis, Tunisia; ^b^ Service de Greffe et Hématologie, Centre National de Greffe de Moelle Osseuse, Tunis, Tunisia; ^c^ faculté de Pharmacie de Monastir, Tunisie; ^d^ Université de Tunis El Manar Faculté de Médecine de Tunis, Tunisie

**Keywords:** Allogeneic stem cell transplantation, complications, cost, pharmacoeconomic

## Abstract

**Background**: Hematopoietic stem cell transplantation (HSCT) is a medically complicated therapy with a long recovery time. In Tunisia, the National Health Insurance Fund (CNAM) covers only the first year post-transplantation, after which the costs are borne by the hospital.

**Objective**: Describe complications that can occur during the second year post-allogeneic HSCT and calculate direct costs in different groups of patients.

**Methods**: In this pharmacoeconomic study, medical records of the second year post-allogeneic HSCT were collected. Studied variables included frequent observed complications and medical and non-medical direct costs.

**Results**: The average total direct cost in the population during the second year post-transplantation was $11,571, 97% of which represents direct medical costs Drugs accounted for the largest share (80%) of total direct costs, dominated by the cost of antifungals (52%) and antivirals (26%) drug . Cytomegalovirus status was seen in 9.3% of patients and was associated with a seven-fold increase in direct costs (*p* < 0.001).​​In patients who developed chronic GVHD, the average direct cost was three times higher than for those who did not (*p* = 0.032).

**Conclusion**: Given the importance of direct costs in the post-transplantation period a review of the hospital financing mechanism and a new convention with the CNAM is crucial.​​

## Introduction

Hematopoietic stem cell (HSC) transplantation is a highly specialized therapy and an expensive medical procedure, which requires important medical resources.

Management of complications, such as infections which can occur during the early phase of neutropenia and late-onset complications following transplantation, in particular graft-versus-host disease (GVHD) [1] and graft rejection, are responsible for increased allograft costs [2,3]. The introduction of new drugs and the use of more specific tools for diagnosis and monitoring have further increased the costs of transplantation.

Several studies have assessed the total cost of allogeneic HSC transplantation [4–6]. However, most of them were limited to pharmacoeconomic aspects of the pre-transplantation treatment analysis, the transplantation procedure, and treatment during the first year after transplantation. Hence, those studies excluded any extra costs going beyond the latter period.

The financing mechanism at the National Bone Marrow Transplant Center of Tunisia [Centre National de Greffe de Moelle Osseuse (CNGMO)] is based on the classification of patients in diagnosis-related groups. A convention has been signed between the CNGMO and the National Health Insurance Fund [Caisse Nationale d’Assurance Maladie (CNAM)], which includes $34,821 limited to the first year post-transplantation. Beyond this time-point, the CNGMO is responsible for all medical fees, except for the so-called specific drugs (immunosuppressive, antiviral, and antifungal drugs)

Thus, the present study, which consists of the estimation of the costs beyond the first year post-transplantation, is part of a national program to help the authorities to revise the reimbursing budget allocated by the CNAM. The aim of the present study is to describe the complications that can occur during the second year after allogeneic HSC transplantation, and to conduct a pharmacoeconomic analysis in different groups of patients according to the risk of disease at transplantation, the source of allogeneic HSC transplant, and resulting complications.

## Materials and methods

### Patients

Our research focused on patients who underwent allogeneic bone marrow (BM) and peripheral stem cell (PSC) transplantation during 2012 because of the availability of data and, above all, to enable the results to be presented to the authorities in order to revise the convention budget with the CNAM. Inclusion criteria were all patients (children and adults) who underwent allogeneic BM or PSC transplantation between 1 January 2012 and 31 December 2012. Exclusion criteria were patients who died during the first year or who were lost to follow-up.

### Study design

This is a retrospective pharmacoeconomic study from perspective of the CNGMO. The study was carried out at the hematology department of CNGMO, starting in January 2015. For all patients, the study started on day 366 post-transplantation and lasted for 1 year.

### Studied variables

#### Patient characteristics

Gender, age, allogeneic HSC transplantation source (BM or PSC), diagnosis, risk of disease at transplantation (high or standard), and death were recorded.

#### Complications in second year post-allogeneic HSC transplantation

The most frequently observed complication in post-allogeneic HSC transplantation patients at the CNGMO were cytomegalovirus (CMV) status (infection and reactivation), and chronic GVHD and relapse.

#### Direct costs

Direct costs represent all the expenses directly attributed to the therapeutic management (medical and non-medical direct costs).

Direct medical costs were calculated by including the total cost per patient for consultations, hospitalization (length of stay, staff costs), drugs, transfusions, drug therapeutic monitoring explorations, and imagery.

Direct non-medical costs were attributed to non-healthcare resources, such as transport, nurses, and equipment used during the post-transplantation period.

#### Data sources

The patients’ characteristics, medical complications, and direct costs were collected from their individual medical records.

Drug costs were calculated using purchasing product prices for 2013 according to the hospital list of drugs. The purchase of drugs is counted on the annual budget of the CNGMO pharmacy.

The costs of biological analyses (virology, bacteriology, hematology, and biochemistry) were defined according to a national hospital codification, where each code is allocated a price.

Data required to estimate the costs of transport and services (equipment, nurses) were gathered via a telephone interview conducted with patients who were still alive. To check the usability of the questionnaire, we carried out a pre-test.​​ This evaluated the clarity and precision of the terms used in the interview, and aimed to avoid ambiguous questions. The number of questions was targeted by avoiding redundancy of similar questions and selecting those that were best suited to this survey. The questions were simple, understandable, and closed.

Costs attributed to the medical and paramedical staff were accounted from the yearly gross salary of staff involved in the treatment adjusted for the number of days spent by each patient in the CNGMO. Costs attributed to hospitalizations were calculated with reference to the annual budget of the center in 2013, granted by the Ministry of Health.

The calculated costs are based on the cost of the US dollar in November 2016 (1 Tunisian dinar = US $2.3).

#### Statistical analysis

To examine the equality of the means of quantitative and qualitative variables, the independent samples *t* test was applied, with equal variances assumed. In this statistical test, a *p* value less than 0.05 was considered significant.

## Results

### Patient characteristics

A total of 45 patients underwent allogeneic HSC transplantation during 2012, of whom 32 were included in this study. The characteristics of the included patients are shown in Table 1.

**Table 1.** Patients’ characteristics and medical conditions.

Characteristic	
Patients, *n*	32
Gender	
Male	21 (66)
Female	11 (34)
Age (years), median (range)	27 (5–50)
HSC transplant source	
BM	18 (56)
PSC	14 (44)
Diagnosed condition	
ALL	9 (28)
AML	9 (28)
CML	3 (9.3)
Fanconi anemia	3 (9.3)
AM	6 (18)
Other (MM, lymphoma)	2 (18)
Risk of disease at transplantation	
High	9 (28)
Standard	23 (72)
Death	2 (6.25)
Complications	
Chronic GVHD	13 (40.62)
CMV status	3 (9.37)
Relapse	1 (3.12)

Data are shown as *n* (%) unless otherwise indicated.

HSC, hematopoietic stem cell; BM, bone marrow; PSC, peripheral stem cell; ALL, acute lymphoblastic leukemia; AML, acute myeloid leukemia; CML, chronic myeloid leukemia; AM, aplasia medular; MM, multiple myeloma; GVHD, graft-versus-host disease; CMV, cytomegalovirus.

### Complications in the second year post-transplantation

The investigation found that 41% of patients who underwent allogeneic HSC transplantation developed chronic GVHD. One patient demonstrated a viral reactivation to CMV and two patients developed CMV disease. Only one patient, who had undergone allogeneic BM transplantation, relapsed from acute myeloid leukemia.

### Total direct costs

The average total direct cost in the population during the second year post-transplantation was $11,571. The median total direct cost was $2448 (Table 2).

**Table 2.** Direct costs ($).

Type of cost	Mean ± SD	(Min–max)	Median
Total direct costs	11,571 ± 20,558	(204–79,443)	2448
Direct costs			
Medical (97%)	11,176 ± 17,443	(186–78,012)	2147
Non-medical (3%)	395 ± 468	(17–1243)	208

Direct costs according to the risk of transplantation and to the post-transplantation complications are reported in Table 3.

**Table 3.** Direct costs ($) in different patient groups.

Risk/type of cost	Mean (min–max)	Median	*p*
Risk of underlying disease at transplantation			
Standard (*n* = 23)	10,335 (204–79,443)	1809	0.546
High (*n* = 9)	14,623 (460–43,012)	16,361	
Chronic GVHD			
Yes (*n* = 13)	19,532 (1738–79,442)	16,361	0.032
No (*n* = 19)	6073 (204–39,993)	1642	
CMV status			
Yes (*n* = 3)	54,149 (39,993–79,443)	43,012	< 0.001
No (*n* = 29)	7133 (204–34,614)	2220	
Relapse			
Yes (*n* = 1)	39,993	–	0.103
No (*n* = 31)	10,623	2373	
Source of HSC transplant			
BM (*n* = 18)	8568 (392–79,443)	1790	0.288
PSC (*n* = 14)	15,362 (204–43,012)	17,757	

GVHD, graft-versus-host disease; CMV, cytomegalovirus; BM, bone marrow; PSC, peripheral stem cell.

Thirteen patients developed chronic GVHD. In this case, the average total direct cost was three times the average of the same cost for patients who did not develop such complication (*p* = 0.032).

Three patients suffered from reactivation of CMV and CMV infection. The average total direct cost for these patients was over seven times the average direct cost for patients who did not suffer from this complication (*p* < 0.001).

In patients at high risk of transplantation disease, the average total cost was almost 1.5 times the average total cost in patients at standard disease risk (Table 3).

#### Direct medical costs

The average direct medical cost was $11,176, which represents 97% of the total direct cost. The median total direct medical cost was $2147 (Table 4). The minimum cost was $186 and the maximum cost was $77,979.

**Table 4.** Direct medical costs ($): costs of different components.

	Mean ± SD	%
Direct medical costs	11,176 ± 17,443	
Medicines	8517 ± 16,848	76.2
Laboratory tests	1542 ± 2657	13.8
Functional explorations	71 ± 146	0.6
Blood products	80 ± 350	0.7
Drug therapeutic monitoring	112 ± 191	1
Hospitalization services	812	7.3
Consultation	42	0.37

**Figure 1.**
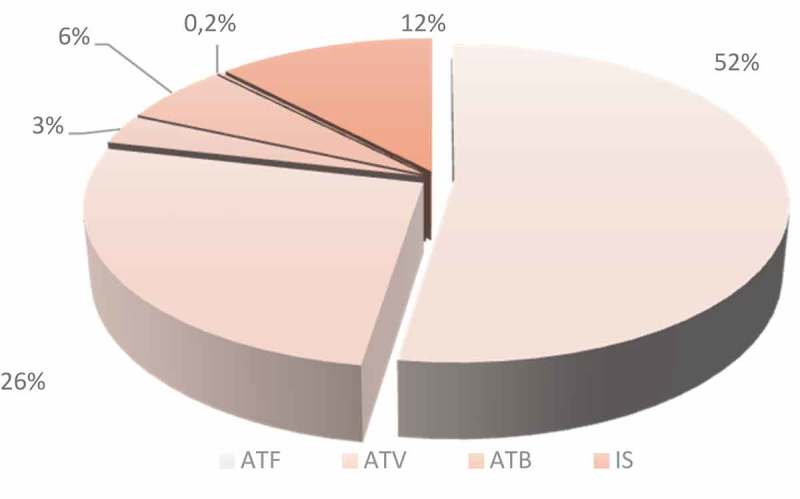
Cost analysis of different therapeutic classes in the second year post-allogeneic hematopoietic stem cell transplantation. ATF, antifungals; ATV, antivirals; ATB, antibiotics; IS, immunosuppressants.​​

The cost of antifungal and antiviral medication was the largest part of the medicines cost, making up 52% and 26% of total drug costs, respectively (Figure 1).

The cost of specific drugs reimbursed by the CNAM represented 67% of total drug costs. The cost of non-specific drugs (provided by the hospital) represented 33% of total drug costs.

#### Direct non-medical costs

The average direct non-medical cost was $395. The average costs of transport and services (equipment and home care) were $247 and $148, respectively.

The average cost of round-trip transportation to the CNGMO for consultation, for patients and their relatives, or to the CNAM for reimbursement, throughout the year, was $247. This represented 65% of direct non-medical costs. The minimum cost of transport throughout the year was $17 and the maximum was $1243.

It should be pointed out that 21% of patients lived in the southern part of the country, 50% in the north, and 29% in the center.

The average cost of services provided by other people, such as nurses, and any special equipment used was $149. This represented 35% of direct non-medical costs (Table 5)

**Table 5.** Direct non-medical costs ($).

	Mean ± SD (min–max)	%
Direct non-medical costs	395 ± 468 (17–1243)	100
Cost of transportation of patients and relatives	247 ± 353	65
Cost of services (equipment and home care)	148 ± 386	35

## Discussion

The main purpose of this pharmacoeconomic analysis was to evaluate the costs directly attributed to therapeutic management during the second year post-allogeneic HSC transplantation. The direct costs were divided into two main classes: direct medical costs (hospitalizations, consultations, drugs, biological tests, drug monitoring costs, etc.) and direct non-medical costs (transport, equipment, and nurses).

The average total direct cost was $11,571, with a median of $2448. Direct medical costs represented 97% of total direct costs, with a mean and median of $11,176 and $2147, respectively. Direct non-medical costs represented only 3% of total direct costs, with a mean and median of $395 and $208, respectively.

A previous study, which evaluated the factors associated with high costs after allogeneic HSC transplantation, reported that increased 1 year costs were seen for post-transplantation complications: rejection [relative hazard (RH) = 1.24, *p* < 0.001], acute GVHD (RH = 1.31, *p* < 0.001), and invasive fungal infection (RH = 1.15, *p* = 0.02) [7].

Our results showed that, for patients who developed chronic GVHD, the average direct cost was three times higher than for those who did not present this complication (*p* = 0.032). The patients with chronic GVHD were treated with immunosuppressive therapy and were therefore more susceptible to the occurrence of potentially severe and life-threatening opportunistic viral and fungal infections.

CMV status is particularly common during HSC transplantation. However, routine monitoring and the use of preventive anti-CMV treatment generally keep this infection under control [8]. CMV status occurred in 9.3% of our patients and was associated with a seven-fold increase in direct costs (*p* < 0.001).​​ A previous study, carried out in Spain, reported that 6% out of 192 patients who were included in the study had developed CMV infection during the third year post-transplantation [9].

In addition to the late onset of infectious complications, relapse of the disease is the second most common cause of increased costs after allogeneic HSC transplantation [10,11]. In our study, only one patient showed a relapse. The direct cost reported was 1.5 times higher than the average direct cost of the rest of the study population.​​

Drug costs represented 76% of direct medical costs, with an average cost of $8517. Biological tests costs represented 14% of direct medical costs, with an average cost of $1542. Other medical parameters (functional evaluation, blood products, plasma drug monitoring, consultation, hospital services) represented a small percentage of direct medical costs.

These results are consistent with a previous study [12], in which the costs of treatment and hospitalization associated with long-term follow-up accounted for almost 60% of the total cost. The average costs (mean ± SD) of treatment and hospitalization in patients who underwent allogeneic BM transplant were $31,566 ± 45,555 and $43,295 ± 57,758, respectively.​​ The total cost was $124,578 ± 147,207.

In the present study, the cost of antifungals (mainly voriconazole) represented 52% of drug costs, followed by antivirals (26%) and antibiotics (12%). Other therapeutic classes, e.g. immunosuppressive drugs and vaccination, represented hardly 10% of the total drug cost.

Some specific drugs, such as voriconazole, valaciclovir, and imatinib, are directly provided by the CNAM, and the hospital did not pay for these treatments.​​ The average cost of specific drugs represented 67% of total drug costs. The non-specific drugs represented 33% of total drug costs. They were funded by the hospital.

Our results show an important variability in the distribution of allograft costs, which is reflected in the standard deviation (SD) values. This variability may be explained by the small number of patients and subgroups, the different diagnoses and conditioning protocols, the different sources of HSC transplants, and the post-transplantation complications.

Others limitations in our study may be considered, such as the non-electronic database. An electronic database system will be necessary to enable the study to be extended for more years, thus enlarging the study population with an equitable distribution between the BM and PSC transplant groups. This will give more rigor and power to future studies.

## Conclusion

In response to a national plan for the revision of the convention budget, we estimated the direct costs of the second year post-transplantation in allogeneic HSC transplantation patients. The results of this study showed that the average direct cost was $11,571, with a minimum of $204 and a maximum of $79,443. A significant difference in costs was associated with post-transplantation complications such as CMV infection and chronic GVHD. All of these costs place a considerable burden on the CNGMO. The authorities should revise the convention budget to cover the years after transplantation, not only the first year, to allow a balanced budget and ensure the viability of the CNGMO.)
